# Mechanisms of the Beneficial Effects of Exercise on Brain-Derived Neurotrophic Factor Expression in Alzheimer’s Disease

**DOI:** 10.3390/biom13111577

**Published:** 2023-10-26

**Authors:** Sama Jaberi, Margaret Fahnestock

**Affiliations:** 1 Graduate Program in Neuroscience, Faculty of Health Sciences, McMaster University, Hamilton, ON L8S 4K1, Canada; jaberis@mcmaster.ca; 2 Department of Psychiatry and Behavioural Neurosciences, Faculty of Health Sciences, McMaster University, Hamilton, ON L8S 4K1, Canada

**Keywords:** brain-derived neurotrophic factor (BDNF), Alzheimer’s disease, exercise, osteocalcin, FNDC5/irisin, lactate, APP processing, dementia, cognition, learning and memory

## Abstract

Brain-derived neurotrophic factor (BDNF) is a key molecule in promoting neurogenesis, dendritic and synaptic health, neuronal survival, plasticity, and excitability, all of which are disrupted in neurological and cognitive disorders such as Alzheimer’s disease (AD). Extracellular aggregates of amyloid-β (Aβ) in the form of plaques and intracellular aggregates of hyperphosphorylated tau protein have been identified as major pathological insults in the AD brain, along with immune dysfunction, oxidative stress, and other toxic stressors. Although aggregated Aβ and tau lead to decreased brain BDNF expression, early losses in BDNF prior to plaque and tangle formation may be due to other insults such as oxidative stress and contribute to early synaptic dysfunction. Physical exercise, on the other hand, protects synaptic and neuronal structure and function, with increased BDNF as a major mediator of exercise-induced enhancements in cognitive function. Here, we review recent literature on the mechanisms behind exercise-induced BDNF upregulation and its effects on improving learning and memory and on Alzheimer’s disease pathology. Exercise releases into the circulation a host of hormones and factors from a variety of peripheral tissues. Mechanisms of BDNF induction discussed here are osteocalcin, FNDC5/irisin, and lactate. The fundamental mechanisms of how exercise impacts BDNF and cognition are not yet fully understood but are a prerequisite to developing new biomarkers and therapies to delay or prevent cognitive decline.

## 1. Alzheimer’s Disease

Alzheimer’s disease (AD) is a neurodegenerative disorder that is clinically characterized by progressive memory loss and cognitive decline and that shows an increasing incidence rate in aging individuals [1]. The 2023 report by the Alzheimer’s Association states that one in three seniors dies with AD or other forms of dementia. Mortality rates due to AD increased 33% for people in the 65 to 74 age group, 51% for the 75 to 84 age group, and 78% for people 85 and older between 2000 and 2019 [1]. Cognitive deficits are characteristic of AD and mild cognitive impairment (MCI), which manifest as impaired memory, attention, and difficulties with executive functions such as problem-solving and decision-making [1]. The decline in cognition worsens as the disease progresses and symptoms become more severe. Of the several pathological hallmarks of AD, extracellular accumulation of amyloid-β (Aβ) aggregates and intracellular aggregates of hyperphosphorylated tau in the form of neurofibrillary tangles are the best-known [2]. Aβ1–42 (Aβ42), the primary component of plaques found in the AD brain, is formed by alternative proteolytic cleavage of the amyloid precursor protein (APP) by the proteases β- (BACE1) and γ-secretases, which then generate a pathological variant of the protein that is soluble and has a tendency to oligomerize [3,4,5]. While extracellular aggregates disrupt synaptic function, intracellular levels of Aβ42 increase in Down syndrome [6] and AD [7,8] and have been linked to apoptotic cell death via a caspase cascade [9,10]. 

The microtubule-associated protein tau assembles microtubules from tubulin and is essential for microtubule stability [11]. Abnormal hyperphosphorylation of tau is observed in AD [12] and is the main component of paired helical filaments and neurofibrillary tangles [12,13,14,15]. In this state, hyperphosphorylated tau does not bind tubulin and disrupts microtubules [16,17]. Hyperphosphorylated tau is prone to aggregation and is toxic to neurons, causing synaptic loss and cognitive impairment. THY-Tau22 mice with mutations in the tau gene show hyperphosphorylation of tau and display deficits in hippocampal synaptic transmission, increased anxiety, delayed learning, and poor memory [18]. 

## 2. Physical Exercise and Brain Health

The benefits of exercise on brain structure and function have been the topic of study for decades. Improvements in synaptic plasticity, structure, and strength with physical exercise training are well-established in the literature [19,20]. In addition to enhanced efficiency in neuronal processing, the prefrontal cortex and hippocampus are larger in adults with higher aerobic fitness [21]. Many studies in animals have shown improved hippocampal-dependent cognitive tasks following a period of exercise participation [19,22,23]. Wheel running improved both spatial and non-spatial memory and learning in rats [22,23] and mice and improved cognitive performance in a mouse model of AD [24].

Human clinical trials on the effects of exercise on cognition have produced variable results. In addition to increases in functional ability, a 6-month aerobic exercise protocol in older adults with AD or MCI showed improved memory performance and reduced hippocampal atrophy [25]. Furthermore, six months of aerobic exercise yielded cognitive enhancement, increased frontoparietal network connectivity, and reduced brain atrophy in patients with Parkinson’s disease (PD) [26]. While the studies above showed slowing of disease progression and reduced brain atrophy in AD, MCI, and PD patients with exercise, another exercise study did not observe any changes in age-related brain atrophy in healthy older adult brains, despite improvements in cognition [27]. However, major limitations of the latter study were the use of the same neurological test forms during initial and follow-up assessments, paired with unsupervised exercise during all but the first few weeks of the study. Therefore, improvements in cognition may have been a practice effect rather than the effect of exercise training [27]. Furthermore, the above results are contradicted by several studies [28,29,30] and meta-analyses [31] that found no significant improvements in executive function, memory, or information processing with exercise training in older adults with subjective cognitive decline, MCI, or dementia, or only reported mild improvements in cognition [32] or improvements in single domains such as executive function [33]. Contradictions in clinical trial results may be explained by differences in protocols such as exercise duration, intensity (low-, moderate-, and high-intensity exercise), and type (aerobic, resistance, and strength exercise). Nevertheless, recent meta-analyses suggest that physical exercise, particularly aerobic exercise, improves cognition in those with MCI and dementia [34]. The mechanism may be by increasing neuroprotective factors such as brain-derived neurotrophic factor (BDNF) [35,36]. Both long-term and acute exercise increase serum and plasma BDNF levels compared to unexercised controls, although regular, long-term exercise results in a higher magnitude of BDNF increase than acute exercise [35]. Furthermore, an evaluation of seven different studies found increased peripheral BDNF levels in individuals with MCI following 8–28 weeks of exercise compared to controls [36]. However, no BDNF effects have been observed with strength exercise [34,35], although the number of studies is low.

## 3. Role of Brain-Derived Neurotrophic Factor

Mechanisms behind exercise-induced improvements in neuronal health, synaptic function, neurogenesis, and cognitive function are currently under investigation. One compelling hypothesis is the role of exercise in regulating growth factors in both central and peripheral tissues. BDNF is a leading candidate for study in this field due to its extensive role in promoting neuronal survival, neurogenesis, synaptic plasticity, and cognitive function [37,38,39,40,41,42]. Voluntary wheel running in rats results in significant increases in BDNF mRNA in the hippocampus, specifically in the dentate gyrus (DG) and Ammon’s horn areas 1 and 4 (CA1 and CA4) [43], CA3, and cerebral cortex [43,44]. Increases in BDNF mRNA are also detected in the spinal cord and skeletal muscle following treadmill training in rats [45].

Measuring BDNF changes in the brains of human participants is not currently feasible; therefore, investigators use BDNF levels in serum or plasma as a proxy. Serum and plasma BDNF levels rise as a consequence of acute or regular exercise [35,46,47,48,49,50]. Peripheral BDNF found in the bloodstream is bound by platelets [51], and although the increased serum or plasma BDNF following exercise is derived mainly from platelets, neurons and vascular endothelial cells also contribute BDNF to both the brain and the bloodstream [52]. However, whether free BDNF can cross the blood-brain barrier bidirectionally and whether peripheral BDNF levels reflect BDNF levels in the brain is controversial. While some studies have shown that brain-derived BDNF can cross the blood-brain barrier and enter the circulation [53,54], other studies demonstrate that circulating BDNF does not enter the brain [55]. Several studies have reported a positive correlation between central and peripheral BDNF levels in animals, suggesting that serum and plasma BDNF levels may reflect brain BDNF levels. A positive correlation was found between hippocampal BDNF levels and plasma BDNF levels in pigs and between BDNF levels in the hippocampus and prefrontal cortex and BDNF levels in the whole blood and serum of rats [56,57,58]. Interestingly, because mouse platelets do not contain BDNF, this neurotrophin has been undetectable in mouse blood [56] until the recent advent of a highly sensitive BDNF ELISA [59]. Consistent with the animal work, human studies of serum or plasma BDNF suggest that exercise-induced increases in peripheral BDNF levels reflect brain BDNF levels. There is evidence of increased serum BDNF levels in younger [48,60] and older adults [46,61,62,63,64] following exercise training. The increased BDNF levels are correlated with higher memory scores as well as increased hippocampal volume post-exercise training, further supporting the usefulness of peripheral measures for judging central effects [60,62,65,66]. 

One of the ways that neurotoxic Aβ42 exerts its neurodegenerative effects is by decreasing BDNF levels and disrupting one of BDNF’s major transcriptional regulators and mediators, cyclic adenosine monophosphate response element binding protein (CREB) [67]. BDNF has a fundamental role in promoting neuronal survival, neurogenesis, maintenance and growth of dendrites, synaptic transmission, plasticity, and excitability [37,67,68,69,70]. Thus, it plays a significant role in hippocampal memory formation. BDNF is involved in the occurrence and maintenance of both early-phase and late-phase long-term potentiation (LTP), which correspond to short-term and long-term hippocampal memory, respectively [71]. 

The severity of cognitive impairments in AD is inversely correlated with the level of BDNF in the brain [72,73]. Significant downregulation of BDNF mRNA, resulting in a 50% reduction of available BDNF protein [72,74], occurs in AD. CREB, a transcriptional regulator of BDNF and a downstream mediator of its activity [75,76,77,78], is also reduced in AD. In glutamate-stimulated hippocampal neurons treated with toxic oligomeric Aβ42, there is a significant decrease in the activity of PKA, which leads to decreased activation of CREB [79]. Oligomeric Aβ42 administration significantly decreases levels of phosphorylated CREB and BDNF mRNA in differentiated SH-SY5Y cells, a human neuroblastoma cell line that displays cortical neuron-like characteristics [80]. Interestingly, in the absence of cell stimulation, Aβ42 downregulates CREB mRNA levels without affecting its basal phosphorylation levels [81]. Therefore, Aβ42 may impair cognitive function by reducing signalling through a BDNF/CREB autoregulatory loop, downregulating BDNF and CREB expression, and reducing CREB phosphorylation.

Pathological tau, or an excess of wild-type tau, also downregulates BDNF at the transcriptional level [82,83]. BDNF mRNA levels are reduced in the postmortem cortical tissue of subjects with tauopathies such as Pick’s disease and corticobasal degeneration in the absence of amyloid pathology compared to healthy controls [82]. BDNF mRNA is also reduced in wild-type tau-overexpressing human neuroblastoma SH-SY5Y cells and in transgenic wild-type tau-expressing mice, either in the presence or absence of neurofibrillary tangles [83]. Furthermore, although BDNF expression is reduced in the APP23 mouse, an amyloid-β model of AD, BDNF levels are partially normalized by crossing these mice with tau knockout mice [83]. Thus, BDNF is downregulated by excess (hyperphosphorylated and/or aggregated) tau even in the absence of mutations or neurofibrillary tangles, and tau is at least partially responsible for mediating amyloid-β-induced BDNF downregulation. Although the precise molecular pathway by which this occurs is not well understood, hyperphosphorylated tau may reduce BDNF expression by inhibiting PKA and CREB phosphorylation [84] or other transcription factors.

## 4. Mechanisms of BDNF Upregulation with Exercise

### 4.1. Physical Exercise Has a Multifaceted Effect on the Body

A diverse array of myokines, cytokines, and peptides are released in response to exercise, which are collectively termed ‘exerkines’ [85]. Many of the myokines released following exercise, such as irisin [86,87], lactate [88,89], cathepsin-B [90], and kynurenic acid [91], upregulate BDNF expression in the hippocampus and enhance cognitive function. In addition, hepatokines such as insulin-like growth factor I (IGF-1) [92,93,94] and fibroblast growth factor 21 (FGF21) [95,96] and other exercise-induced molecules such as osteocalcin [97] and β-hydroxybutyrate [98] also enhance cognition by upregulating BDNF. This review will focus on three of the above exerkines: osteocalcin, irisin, and lactate, due to an increase in recent research on these factors. We will outline recent literature on the proposed mechanisms by which each of these molecules upregulates BDNF and enhances cognitive function.

### 4.2. Osteocalcin, a Bone-Derived Hormone, Plays a Role in Learning and Memory via BDNF

**Figure 1:**
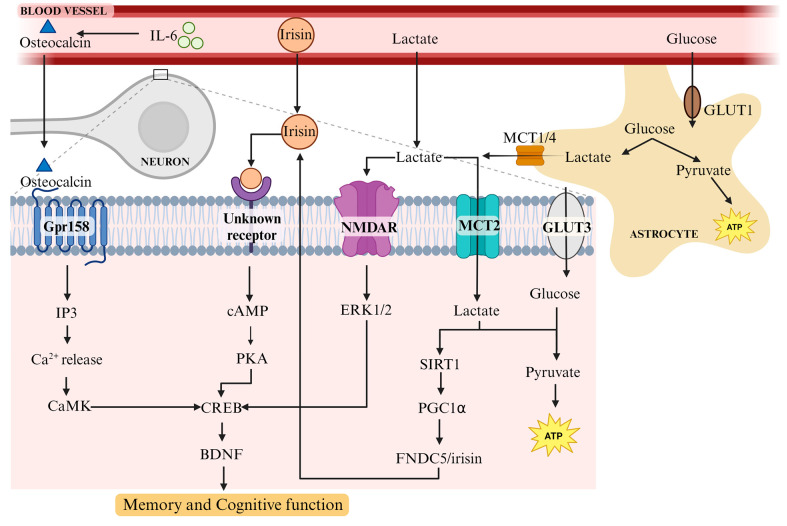
Role of osteocalcin, FNDC5/irisin, and lactate in BDNF upregulation and enhanced memory and cognitive function in response to exercise. Following exercise, osteocalcin is released from osteoblasts, irisin and lactate are released from muscle, and lactate, a product of glucose metabolism, is released by CNS astrocytes. Grey dotted lines indicate enlargement of the boxed area. Osteocalcin/Gpr158 signals through IP3, CaMK, and CREB to upregulate BDNF. Irisin activates CREB through PKA to upregulate BDNF. Lactate is transported into neurons through MCT2 and increases FNDC5/irisin expression, feeding into the irisin pathway. In addition, lactate acts through NMDA receptors to activate ERK1/2 and CREB, upregulating BDNF. Ultimately, BDNF upregulation enhances memory and cognitive performance. IL-6: interleukin-6; GLUT1/3: Glucose transporter1/3; ATP: Adenosine triphosphate; MCT1/2/4: Monocarboxylate transporters 1/2/4; GPR158: G protein-coupled receptor 158; NMDAR: N-methyl-D-aspartate receptor; IP3: Inositol triphosphate; Ca^2+^: Calcium; CaMK: Ca^2+^/calmodulin-dependent protein kinase; cAMP: Cyclic adenosine monophosphate; PKA: Protein kinase A; CREB: cAMP-response element binding protein; ERK1/2: Extracellular signal-regulated kinase 1/2; SIRT1: Silent information regulator 1; PGC1α: Peroxisome proliferator-activated receptor-γ coactivator; FNDC5: Fibronectin type III domain-containing protein 5; BDNF: Brain-derived neurotrophic factor. Created in Biorender.

Osteocalcin, an osteoblast-specific hormone, functions in an array of physiological processes such as glucose homeostasis and exercise capacity, as well as brain development and cognition [99]. One session of high-intensity exercise in human participants produces a significant increase in serum levels of uncarboxylated osteocalcin (a bioactive form of osteocalcin) [48,100,101]. Release of uncarboxylated osteocalcin from bone is induced by interleukin 6 (IL-6), an anti-inflammatory myokine secreted from muscle in response to exercise [102] (Figure 1). IL-6 signalling induces osteoclast differentiation and promotes uncarboxylated osteocalcin release. This is evident in IL-6-deficient mice that do not exhibit an increase in osteocalcin levels in response to exercise [102].

Several studies have suggested that osteocalcin plays a role in mood and cognition. In middle-aged and older women, higher osteocalcin levels are associated with better executive function and cognitive performance [97,103]. Administration of osteocalcin in the APP/PS1 AD mouse model enhances spatial learning, memory, and glial glycolysis and reduces anxiety, Aβ42, and gliosis [104]. Osteocalcin knockout mice exhibit impaired spatial learning and hippocampal-dependent memory, as well as increased anxiety-like behaviour [105]. Moreover, osteocalcin knockout mice have smaller brains than wild-type mice, with particular deficits seen in the dentate gyrus and corpus callosum [106]. Injecting plasma from young mice into older mice improved age-related cognitive impairment [107], but only when the plasma contained osteocalcin [108]. Plasma from osteocalcin knockout mice improved hippocampal-dependent memory and reduced anxiety-like behaviour in aged wild-type mice only when supplemented with uncarboxylated osteocalcin.

Osteocalcin’s effects on cognition may be mediated by BDNF. Khrimian et al. [108] identified an orphan class C G-protein-coupled receptor (GPCR), Gpr158, as the receptor through which osteocalcin mediates its cognitive effects on the brain (Figure 1). In a signalling pathway assay, osteocalcin was found in a complex with Gpr158/Gαq and increased the production of inositol 1,4,5-triphosphate (IP3) in wild-type hippocampal neurons. IP3 is a second messenger that facilitates the exocrine secretion of various molecules, including BDNF, and may also mediate increases in BDNF expression [109]. Binding of IP3 to its receptor causes a surge of calcium into the cytoplasm from intracellular calcium stores [109], which leads to the activation of a Ca2+/calmodulin-dependent kinase (CaMK) pathway that upregulates CREB [78,110]. The role of osteocalcin/Gpr158 signalling in BDNF expression is evident in Gpr158-knockout mice, where BDNF expression is significantly reduced, and while osteocalcin treatment does not alter BDNF expression in these mice, it significantly increases BDNF expression in wild-type mice expressing Gpr158 [108]. This confirms that osteocalcin increases BDNF expression in the brain via the Gpr158 receptor.

**Figure 2:**
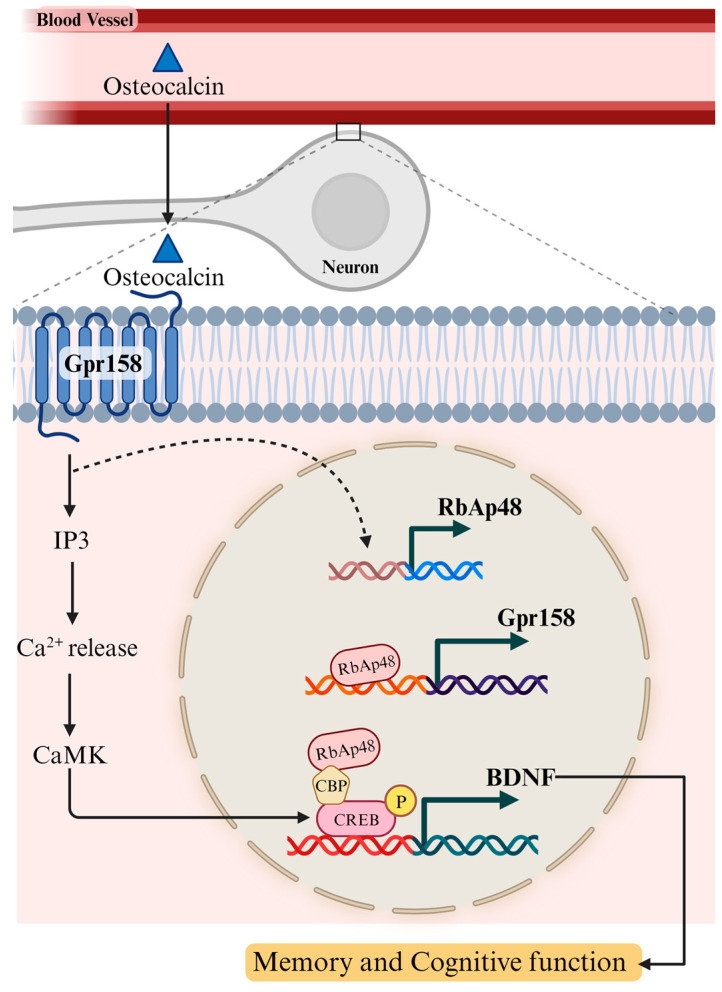
RbAp48 regulates osteocalcin/Gpr158 signalling. Uncarboxylated osteocalcin crosses the blood-brain barrier and activates its receptor in the brain, Gpr158. The histone-binding protein RbAp48 is involved in the transcriptional activation of Gpr158, while Gpr158 signalling also modulates RbAp48 expression via an unknown mechanism (depicted by the dotted arrow), creating a feedback loop. RbAp48 is also found in a complex with CBP and CREB, which activates BDNF expression. Grey dotted lines indicate enlargement of the boxed area. GPR158: G protein-coupled receptor 158; RbAp48: Retinoblastoma binding protein 4; BDNF: Brain-derived neurotrophic factor; IP3: Inositol triphosphate; Ca^2+^: Calcium; CaMK: Ca^2+^/calmodulin-dependent protein kinase; P: Phosphate; CBP: CREB-binding protein. Created in BioRender.

Another important component of osteocalcin/Gpr158 signalling is the histone-binding protein, RbAp48 [111] (Figure 2). RbAp48 can be found in a complex with CREB binding protein (CBP) and enhances its acetyl transferase activity [112], which is critical for long-term memory formation and synaptic plasticity [113,114,115]. Recently, RbAp48 was also found to be involved in transcriptional activation of Gpr158 by binding to its promoter, controlling its expression in the hippocampus. In turn, osteocalcin/Gpr158 signalling modulates RbAp48 expression, since knocking down Gpr158 leads to a reduction in RbAp48 protein levels and memory performance, while activation of the osteocalcin/Gpr158 pathway upregulates RbAp48 expression and rescues age-related memory loss [111].

Current literature demonstrates that physical exercise increases uncarboxylated osteocalcin levels, which act through Gpr158 to improve hippocampal memory. Evidence suggests that osteocalcin’s effects are mediated by BDNF. Further questions include whether osteocalcin improves cognitive function in the absence of BDNF signalling and the role of RbAp48 in osteocalcin-induced BDNF upregulation.

### 4.3. FNDC5/Irisin Increases BDNF Levels in the Hippocampus and Supports Learning and Memory

Exercise induces the expression of many distinct types of proteins, including several active myokines from muscle. The muscle-derived factor, irisin, is released into the circulation immediately after exercise [86] (Figure 1). Irisin is released by proteolytic cleavage of the extracellular portion of the fibronectin type III domain containing 5 (FNDC5), a transmembrane glycoprotein [86]. Irisin functions in energy expenditure, metabolism, and insulin resistance. In addition to its roles in metabolism, FNDC5/irisin is also expressed in the hippocampus and cortex [87,116]. Irisin influences neuronal development, since knocking down FNDC5 in mouse embryonic stem cells significantly reduces their neuronal differentiation [117]. FNDC5/irisin is reduced in AD and in AD mouse models, leading to impaired long-term potentiation and poor memory performance. Conversely, overexpression of FNDC5 in an AD mouse model rescues memory and synaptic plasticity [87]. Furthermore, exercise prevents reductions in FNDC5/irisin, BDNF, and memory performance in mouse models of AD.

Peripheral delivery of irisin increases central levels of irisin in FNDC5 knockout mice and in mouse models of AD and rescues cognitive impairment in these mice [87,118]. Interestingly, inhibition of either peripheral or central FNDC5/irisin leads to impaired long-term potentiation and poor memory performance in exercised mice [87].

The neuroprotective effects of irisin are mediated by increases in BDNF expression. Exercise-induced expression of FNDC5 in the hippocampus stimulates the expression of BDNF in the brain, but not in brain areas where FNDC5 is not expressed [116]. Expression of FNDC5 in primary cortical neurons increases expression of BDNF and mediators of BDNF involved in hippocampal function, including Npas4, c-Fos, and Arc [116]. Treatment of mouse hippocampal and human cortical slices with recombinant irisin stimulates the cAMP/PKA/CREB pathway [87], which is known to increase BDNF mRNA and protein levels [67] (Figure 1). Conversely, BDNF expression is reduced in primary cortical neurons treated with FNDC5 shRNA and in peroxisome proliferator-activated receptor-gamma coactivator (PGC)-1α knockout mice, a key regulator of FNDC5 gene expression [116,119]. Mice subjected to treadmill running exhibit increases in hippocampal PGC1α and FNDC5 protein levels and plasma irisin levels, which correlate with increased BDNF mRNA levels and cell proliferation in the hippocampus. These increases are blocked when the mice are treated peripherally with an irisin-neutralizing antibody [120]. Lastly, peripheral expression of FNDC5 in the liver via adenoviral vectors increases BDNF expression in the brain, suggesting that peripheral irisin or a metabolite may cross the blood-brain barrier and induce BDNF expression [116]. Whether the main effect of exercise-induced irisin on cognition is via peripheral or central irisin is not fully understood. The mechanism and transporters have not been identified. Regardless, the above results confirm that exercise-induced irisin has a direct effect on BDNF expression levels in the hippocampus and plays a critical role in spatial learning and memory.

### 4.4. Lactate Release from Muscle following Exercise Induces BDNF in the Hippocampus and Promotes Learning and Long-Term Memory Formation

An important byproduct of exercise is lactate released by exercising muscles, which is commonly known to cause a burning sensation during intense exercise. When oxygen becomes limited in muscle cells, pyruvate is reduced in a reaction catalyzed by lactate dehydrogenase, producing lactic acid. During this process, nicotinamide adenine dinucleotide (NAD+) is regenerated, allowing glycolysis to continue to generate adenosine triphosphate (ATP) in the absence of oxygen. For many years, lactate was overlooked as only a byproduct. However, recent evidence demonstrates the beneficial role of lactate in the body and the brain.

Peripheral lactate can be transported across the blood-brain barrier via monocarboxylate transporter 1 (MCT1) [121,122], but lactate in the brain is predominantly generated from glucose metabolism in astrocytes [123,124] (Figure 1). During excitatory neurotransmission, lactate provided to neurons by astrocytes can be used as an energy source by conversion to pyruvate, which enters the citric acid cycle [125]. However, it has also been suggested that astrocyte-neuron lactate transport is necessary for long-term memory formation [123,126] and can regulate memory processing [127]. Increased lactate levels in the cerebrospinal fluid are found in the early but not later stages of MCI and AD [128,129]. Knocking down lactate transporters MCT1 and MCT4 (expressed in astrocytes and oligodendrocytes) in the rat hippocampus disrupts long-term but not short-term memory. Exogenous administration of L-lactate, but not glucose, rescues memory retention in these MCT knockdown rats [123]. Interestingly, knocking down MCT2 (expressed in neurons) also disrupts long-term memory formation, but L-lactate and glucose administration fail to rescue memory retention in these rats. This confirms that lactate transport to neurons through MCT2 is necessary for long-term memory formation.

There is a positive correlation between peripheral circulating lactate concentrations and BDNF levels in human blood, since intravenous lactate infusion elevated blood BDNF levels in young adults [130]. Two potential mechanisms through which lactate might increase BDNF expression are by potentiating N-methyl-D-aspartate receptor (NMDAR) signalling [88] and by activating the PGC1α/FNDC5/BDNF pathway [89] (Figure 1). Yang et al. (2014) [88] showed that L-lactate induces the expression of plasticity-related genes such as Arc, Zif268, c-Fos, and BDNF in neurons *in vitro* and *in vivo*. Lactate may increase BDNF expression by activating NMDA receptors and extracellular signal-regulated kinase 1/2 (Erk1/2). When treated with an NMDAR antagonist, increases in Arc and Zif268 mRNA and protein levels [88] and BDNF mRNA levels [131] were blocked. Inhibiting Erk1/2 reduced L-lactate-stimulation of Arc and Zif268 mRNA and protein levels, although the effect of blocking Erk1/2 on BDNF and c-fos expression was not reported in this study. However, it is known that Erk1/2 can activate CREB and upregulate BDNF expression as a result [132], and that BDNF can activate CREB and Erk1/2 [70]. Together, these results confirm that lactate induces an increase in plasticity-related genes via the Erk1/2 pathway, which is downstream of NMDAR signalling [133]. These effects were not observed with D-lactate (its nonmetabolized enantiomer), L-pyruvate, or D-glucose, making them selective for L-lactate.

El Hayek et al. (2019) [89] demonstrated that hippocampal increases in BDNF expression and signalling in exercising mice are lactate-dependent. Intraperitoneal injection of an MCT1/2 inhibitor abolished the increase in BDNF expression and signalling in exercising mice, showing that lactate transport to neurons via MCT1/2 is necessary for exercise-induced BDNF expression and signalling. Moreover, lactate significantly increased BDNF expression in hippocampal and cortical neurons in culture. Lactate-injected mice exhibited better performance than control mice in Morris Water Maze tests of spatial learning and memory, but not when cotreated with the tyrosine kinase inhibitor CEP701, which inhibits Trks. Thus, these results indicate that lactate may increase BDNF signalling to improve learning and memory. Voluntary exercise or lactate injections increased hippocampal PGC1α and FNDC5 protein levels, suggesting that lactate-induced BDNF expression may occur through the PGC1α/FNDC5/BDNF pathway (Figure 1). Furthermore, both voluntary exercise and intraperitoneal lactate injections led to an increase in hippocampal levels of silent information regulator 1 (SIRT1), which is a NAD+-dependent deacetylase involved in gene regulation [125]. Knocking down SIRT1 abolished lactate-induced BDNF expression in the hippocampus. Moreover, cotreatment with lactate and an inhibitor of SIRT1, sirtinol, blocked increases in PGC1α protein levels [89]. Therefore, lactate leads to hippocampal expression of BDNF through a SIRT1-dependent induction of PGC1α and FNDC5. As mentioned previously, the PGC1α/FNDC5/BDNF pathway activates the cAMP/PKA/CREB pathway, which upregulates BDNF expression and regulates learning and memory formation.

## 5. Exercise-Induced BDNF Reduces APP Toxicity by Altering Its Processing

Inactivity is a risk factor for AD [134,135,136,137,138]. Of note are the higher levels of circulating Aβ protein in sedentary individuals compared to individuals who exercise [137,138,139] and the correlation between increased circulating Aβ and an increased risk of developing AD and MCI [5]. Both *in vitro* and *in vivo* studies show that BDNF reduces amyloidogenic Aβ and decreases its neurotoxic effects [140,141,142], and it may mediate this effect by altering APP processing. Therefore, investigators explored a possible relationship between BDNF and some of the enzymes involved in APP processing: α-, β-, γ-, and δ-secretase. α-secretase is involved in non-amyloidogenic cleavage of APP, while β- and γ-secretase promote the production of toxic Aβ [143]. δ-secretase, also known as asparagine endopeptidase (AEP), is a cysteine proteinase activated during aging that cleaves APP. δ-secretase cleavage generates an APP fragment that may be the preferred substrate for β-secretase, thereby enhancing β-secretase cleavage of APP and the production of Aβ [144]. In the absence of BDNF signalling, δ-secretase expression is increased via C/EBPβ upregulation [142], while increased BDNF signalling increases Akt phosphorylation of δ-secretase, which inhibits its activity [145].

In parallel, β-site amyloid precursor protein cleaving enzyme 1 (BACE1 or β-secretase) is an important enzyme responsible for cleaving APP and releasing soluble Aβ peptide (i.e., amyloidogenic pathway). There is evidence that both chronic and acute exercise in mice lead to a reduction of BACE1 content, consequently decreasing Aβ accumulation and improving recognition memory [146,147]. Treatment of brain tissue with BDNF yielded a significant reduction in BACE1 activity [146], while BDNF deprivation yielded increased BACE1 protein levels [142]. These results suggest another possible mechanism by which BDNF reduces amyloidogenic APP processing, although more studies are needed to elucidate the mechanism of BDNF-induced BACE1 downregulation.

A third possible mechanism by which exercise-induced BDNF reduces Aβ production is by enhancing ADAM10 activity. ADAM10 is the main active component of α-secretase, which processes APP to generate and secrete nonamyloidogenic APPα [148]. As expected, treatment of differentiated human SH-SY5Y neuronal cells with an ADAM10 inhibitor significantly increased the production of Aβ. BDNF treatment of SH-SY5Y cells significantly reduced Aβ production. However, when cotreated with BDNF and ADAM10 inhibitors, Aβ levels were still significantly higher than controls [149]. These results suggest that BDNF reduces Aβ toxicity by enhancing ADAM10 activity, since inhibition of ADAM10 abolished BDNF’s effect on reducing Aβ production. Interestingly, BDNF does not alter ADAM10 protein levels [147,149] but enhances its activity by altering its distribution in the cell toward intracellular accumulation, where regulated α-secretase activity occurs, rather than on the cell surface [149]. Together, these results suggest that possible mechanisms by which exercise-induced upregulation of BDNF reduces AD pathology include increasing α-secretase activity and decreasing β- and δ-secretase levels, shifting the balance of APP processing towards the nonamyloidogenic pathway, and reducing Aβ toxicity in the brain.

## 6. Conclusions

BDNF is a critical molecule for neuronal health and survival, neurogenesis, synaptic plasticity, neuronal excitability, and learning and memory. BDNF deficiency is correlated with mild cognitive impairment, Alzheimer’s disease, Parkinson’s disease, and other neurodegenerative disorders. Physical exercise, particularly aerobic exercise, has produced promising results in improving neurodegenerative- and age-related learning and memory deficits, and its effects are at least partially mediated by upregulating BDNF levels. Here, we reviewed several molecules released in response to exercise that facilitate BDNF upregulation. Uncarboxylated osteocalcin release, induced by IL-6, increases BDNF expression (Figure 1). Osteocalcin-induced upregulation of BDNF is mediated through Gpr158 signalling, which activates a pathway involving IP3/CaMK/CREB. Another exercise-induced molecule is FNDC5/irisin, which is expressed in the brain and released from muscle post-exercise. Peripheral and central irisin both contribute to learning and memory by stimulating the cAMP/PKA/CREB/BDNF pathway. Future studies should investigate the transporters and mechanisms by which irisin may cross the blood-brain barrier. Moreover, it is important to consider whether peripheral or central irisin is responsible for its effects on cognition post-exercise. Similarly, lactate is released from muscle following exercise and is also produced by astrocytes in the brain. It may upregulate BDNF expression in the brain by activating NMDA receptors and also by stimulating the FNDC5/irisin pathway. Interestingly, lactate-induced induction of the PGC1α/FNDC5/BDNF pathway appears to be dependent on SIRT1, although more investigation is required to confirm this. Exercise-induced BDNF upregulation reduces amyloidogenic Aβ levels. Mechanisms behind this reduction may be achieved by reducing amyloidogenicity and promoting nonamyloidogenic cleavage of APP. Physical exercise is a cost-effective intervention that has demonstrated favourable outcomes in improving cognitive impairment in neurodegenerative diseases such as Alzheimer’s disease. Reviewed here are a few of the many mechanisms and molecules involved in the beneficial effects of exercise on the brain. A comprehensive understanding of these mechanisms is key to developing biomarkers and therapeutics to slow cognitive decline.

## Data Availability

No new data were created or analyzed in this study. Data sharing is not applicable to this article.
